# The Role of Fecal Microbiota Transplantation (FMT) in the Management of Metabolic Diseases in Humans: A Narrative Review

**DOI:** 10.3390/biomedicines12081871

**Published:** 2024-08-16

**Authors:** Eva Zikou, Chrysi Koliaki, Konstantinos Makrilakis

**Affiliations:** First Department of Propaedeutic Internal Medicine and Diabetes Center, Medical Faculty, National and Kapodistrian University of Athens, “Laiko” General Hospital, 17 Agiou Thoma Street, 11527 Athens, Greece; zikoudiatrofi@gmail.com (E.Z.); kmakrila@med.uoa.gr (K.M.)

**Keywords:** fecal microbiota transplantation, gut microbiota, insulin resistance, metabolic syndrome, obesity, type 2 diabetes mellitus

## Abstract

The gut microbiota represents a complex ecosystem of trillions of microorganisms residing in the human gastrointestinal tract, which is known to interact with the host physiology and regulate multiple functions. Alterations in gut microbial composition, diversity, and function are referred to as dysbiosis. Dysbiosis has been associated with a variety of chronic diseases, including *Clostridioides difficile* infections, but also cardiometabolic diseases, including obesity, metabolic syndrome, and type 2 diabetes mellitus (T2DM). The implication of gut microbiota dysbiosis in the pathogenesis of both obesity and T2DM has paved the way to implementing novel therapeutic approaches for metabolic diseases through gut microbial reconfiguration. These interventions include probiotics, prebiotics, and synbiotics, while a more innovative approach has been fecal microbiota transplantation (FMT). FMT is a procedure that delivers healthy human donor stool to another individual through the gastrointestinal tract, aiming to restore gut microbiota balance. Several studies have investigated this approach as a potential tool to mitigate the adverse metabolic effects of gut microbiota aberrations associated with obesity and T2DM. The aim of the present review was to critically summarize the existing evidence regarding the clinical applications of FMT in the management of obesity and T2DM and provide an update on the potential of this method to remodel the entire host microbiota, leading thus to weight loss and sustained metabolic benefits. Safety issues, long-term efficacy, limitations, and pitfalls associated with FMT studies are further discussed, emphasizing the need for further research and standardization in certain methodological aspects in order to optimize metabolic outcomes.

## 1. Introduction

Metabolic diseases, such as obesity and type 2 diabetes mellitus (T2DM), represent serious public health issues with escalating global prevalence trends. They are complex diseases caused by a variety of genetic, epigenetic, and environmental factors [1,2]. Obesity is a multifactorial, difficult-to-treat, and often relapsing chronic disease, associated with substantial morbidity and mortality, ranging from premature death to chronic debilitating conditions including T2DM, cardiovascular diseases, and malignancies [2,3]. Obesity has been consistently associated with chronic low-grade inflammation, induced in response to excess nutrient availability [4,5]. Obesity-associated inflammation may contribute among others to the pathogenesis of T2DM, which is a strongly interrelated disease [6]. T2DM is characterized in pathophysiological terms by pancreatic β cell dysfunction leading to progressively reduced insulin secretion and peripheral tissue insulin resistance, both induced by mechanisms related to glucotoxicity, lipotoxicity, subclinical inflammation, and oxidative stress [7]. The cornerstone of management of both obesity and T2DM is lifestyle modification, by means of reducing the energy density and improving the quality of caloric intake as well as increasing physical activity, complemented by specific pharmaceutical therapeutic approaches targeted mainly at appetite suppression, if clinically indicated [8,9].

The gut microbiota represents a complex ecosystem of several trillions of microorganisms (bacteria and fungi) residing in the human gastrointestinal tract, which is known to interact with the host physiology and regulate multiple functions [10,11,12,13]. It develops mostly during a critical period in early childhood and can be altered by antibiotic exposure, Caesarean sections, and diet-related factors. Alterations in gut microbial composition and function, and specifically an imbalance in gut microbiota composition, diversity, and functional capacity, are commonly referred to as “dysbiosis”. This term indicates a shift in gut bacterial communities, as compared to healthy individuals, towards an unbalanced composition being often enriched with pro-inflammatory microbes such as Proteobacteria and mostly characterized by reduced diversity and decreased levels of beneficial metabolites such as short-chain fatty acids (SCFAs) [14,15,16]. Dysbiosis has been associated with a variety of human chronic diseases, both digestive and extra-digestive, including *Clostridioides difficile* infections (CDI), irritable bowel syndrome (IBS), inflammatory bowel disease (IBD), and cardiometabolic diseases including obesity, metabolic syndrome, and T2DM [17,18,19]. Interestingly, metabolic diseases, and especially diabetes, have been associated with adverse outcomes in gastrointestinal conditions characterized by compromised microbiota balance such as IBD, as concluded in a recent systematic review and meta-analysis, showing a negative impact of diabetes on IBD course by increasing the risk of hospitalizations and infections [20].

Gut microbiota changes have been related to obesity pathophysiology, since gut bacteria of obese individuals have been associated with an increased production of SCFAs, which provide additional calories to the host, thus promoting weight gain, and further stimulate the expression of receptors involved in adipogenesis [21,22]. Multiple studies have reported specific perturbations in the gut microbiota of obese compared to normal-weight humans [23,24,25]. In the same direction, the stability, abundance, and qualitative composition of gut microbiota have been linked to the development of T2DM and its associated complications, as patients with T2DM have distinctly different gut microbiota compared to non-diabetic individuals [26]. Studies have shown an increase in opportunistic pathogens and a decrease in butyrate-producing bacteria in patients with T2DM, substantiating the concept of gut microbiota dysbiosis in the setting of metabolic diseases [27,28].

The implication of gut microbiota dysbiosis in both obesity and T2DM pathogenesis has paved the way to implementing novel treatment approaches for metabolic diseases through gut microbial reconfiguration. Several therapeutic strategies have been developed to re-establish intestinal eubiosis. These include the administration of probiotics [29,30,31,32,33,34], prebiotics [35,36,37,38], and the combined administration of probiotics and prebiotics (synbiotics) [38,39,40,41], while a more recent innovative approach has been fecal microbiota transplantation (FMT) [42]. FMT, also known as stool transplantation, is a procedure that delivers healthy human donor stool to another individual through the gastrointestinal tract, aiming to restore the balance of gut microbiota, and several studies have investigated this approach as a potential tool to mitigate the adverse metabolic effects of gut microbiota aberrations associated with obesity and T2DM [43,44]. The key mechanisms underlying the modification of human physiology induced by FMT, especially in the context of metabolic improvement, comprise the following: increased microbial diversity, altered composition of microbial metabolites acting on the host metabolism and immune system, altered gut permeability, and altered gut–brain axis leading to changes in mood and eating behavior [45]. Besides targeting gut bacteria, it has been shown that modifying the gut microbiota by introducing pro- and prebiotic fungi can reduce intestinal inflammatory burden, which might have important implications not only for patients with IBD, but also in the setting of metabolic diseases, considering that a pro-inflammatory intestinal microenvironment has been associated with obesity and diabetes within a bidirectional cause–effect relationship. Experimental ex vivo studies in inflamed mucosa tissue of patients with IBD have yielded interesting results, showing a down-regulation of pro-inflammatory and an up-regulation of anti-inflammatory cytokine expression with the administration of a nutraceutical compound composed of *Hericium erinaceus*, berberine, quercetin, biotin, and niacin [46].

The aim of the present narrative review was to critically summarize the existing scientific evidence regarding the clinical applications of FMT in the management of obesity and T2DM and provide an update on the potential of this method to resolve gut microbiota abnormalities observed in metabolically compromised individuals and remodel the entire host microbiota, thus leading to weight loss and sustained metabolic benefits. Furthermore, we discuss safety, long-term treatment efficacy mainly depending on engraftment success, potential limitations, and pitfalls associated with FMT studies, emphasizing the need for further research and standardization in certain methodological aspects in order to optimize outcomes. 

## 2. Methods of Literature Search and Review Criteria

For the preparation of this review, we applied the following search terms “fecal microbiota transplantation”, “gut microbiota”, “gut dysbiosis”, “intestinal flora”, “metabolic diseases,” “obesity”, “metabolic syndrome”, “insulin resistance”, and “type 2 diabetes mellitus” in all possible combinations in order to retrieve the available scientific literature data from PubMed, Medline, and Google Scholar electronic databases from inception until June 2024. The literature search included papers written in the English language that involved systematic reviews, meta-analyses, and clinical intervention trials in humans, and additional references were retrieved from reviewing the references cited in the original articles.

## 3. Underlying Principles and Delivering Methods of FMT

The human gut microbiota is composed by approximately 10^12^ microorganisms that belong to the community of bacteria, archaea, fungi, and others, and this complex ecological community of intestinal flora has co-evolved with its host as its abundance may relate positively or negatively to several diseases [47,48].

FMT refers to the therapeutic procedure of transplanting fecal bacteria from healthy donors into patients, aiming to restore microbial diversity and decrease the abundance of unfavorable microbial genera, leading thus to a balanced gut microbiota profile and treatment of diseases associated with gut microbiota alterations [44,49]. FMT transfers not only bacteria, but the entire microbiota, comprising also human proteins and/or donor cells. More analytically, microbiota account for 55% of transplanted fecal material in usual FMT, while soluble components including mucus, proteins, fat, bile acids, SCFAs, small-molecule metabolites, and colonocytes account for 24% [45]. The transplanted feces also contain a considerable amount of human DNA, presumably from excreted epithelial and immune cells. 

The heterologous transplantation of material derived from donors’ stool is called allogenic FMT (Allo-FMT) [50]. As opposed to Allo-FMT, autologous FMT (Auto-FMT) refers to the collection of an individual’s own microbiota and transplanting it back to the same person and has gained considerable scientific attention based on its potential to reestablish the original gut microbiota through beneficial alterations in microbial diversity and composition [51]. Auto-FMT has important advantages over Allo-FMT, as it decreases the risk of donor-acquired infections and increases treatment efficacy [52]. The Auto-FMT has been applied in allogenic hematopoietic stem cell transplantation, in order to reduce the gastrointestinal side effects caused by antibiotics [53], and has been further tested in the management of several gastrointestinal diseases [52,54]. 

The FMT procedure includes three key stages, which comprise donor selection, preparation of fecal material, and delivery [55]. In the case of Allo-FMT, the donor selection takes place under close scrutiny in several stages, in order to prevent adverse events related to the infused fecal material. Donor stool can be provided either from patient-related donors or from universal anonymous donors via stool banks, which is usually preferred due to ethical and cost issues [56,57,58]. The process of appropriate donor selection involves several stages and requirements for a potential donor to be accepted, including a preliminary interview, a pre-screen survey, as well as stool and blood examinations, aiming to preclude the presence of infectious diseases that could be potentially transmitted to the recipient [59]. Healthy individuals aged 18–60 years old with a normal body mass index (BMI) are preferred [60], while the detailed exclusion criteria, summarized in Table 1 and Table 2, demonstrate the strict requirements set by the European Commission for the selection of allogenic living donors of human tissue transplants [59]. Currently, blood screening protocols typically include a complete blood count, liver function tests, human immunodeficiency virus (HIV), cytomegalovirus (CMV), Epstein–Barr virus (EBV), syphilis, and hepatitis detection tests, while the stool screening components encompass polymerase chain reaction (PCR) and immunoassay tests for *Clostridioides difficile* toxins, *Cyclospora*, *Isospora, Cryptosporidium, Giardia lamblia,* and *Helicobacter pylori* antigens [61]. 

When the screening of the candidate donor is completed, the fecal sample is obtained and the preparation of the fecal transplant begins, either for delivery directly to the recipient or for cryopreservation [62,63]. One commonly used protocol for sample extraction is the Amsterdam protocol, which uses an amount of 200–300 g of stool dissolved in 500 mL of a sterile saline solution within 6 h of sample collection [64]. It should be noted that the microbial content of stool samples is absolutely diverse between different subjects and even between different donations [65]. Studies have shown that approximately 30–60 g of fecal material may be sufficient for the procedure of FMT [57,66]. However, the stool weight is not necessarily related to microbiota quantity, as there can be significant qualitative alterations of microbial composition between donors and even between donors’ several donations [56,60,67]. After extraction, the sample is frozen until its transplantation. Stool storage has several limitations [68], including the homogenization of fresh stool with normal saline solution, the removal of insoluble particles through laboratory sieves, the centrifugation, the modification with sterile pharmaceutical-grade glycerol for cryoprotection, and finally freezing at −80 °C [69,70,71]. Although prolonged freezing of fecal samples at −80 °C seems to preserve their microbial composition, its effect on viability remains unclear. Furthermore, there has been some debate on whether the use of fresh vs. frozen fecal samples may impact the overall treatment efficacy. Although, according to the literature, both fresh and frozen samples have yielded similar results [72], frozen samples remain the preferred modality for practicality reasons [71].

The final stage of transplant delivery begins with the preparation of the recipient. The reduction in the recipient’s commensal flora is typically achieved with a multi-dose antibiotic regimen of vancomycin or doxycycline [73]. The aim of this pre-procedural antibiotic treatment is to eliminate the current strains harbored in the recipient, so that the newly transplanted bacteria can survive without competition for space and resources. Along with the antibacterial treatment, the host is often given a polyethylene glycol laxative, which has been shown to enhance the colonization of the newly transplanted bacteria [74]. Once the recipient’s gut is cleansed of the thousands of preexisting bacterial colonies, the donor gut microbiota is ready to be transplanted. There are several routes of fecal administration that have been used in clinical research and practice. Delivering ways for FMT may include the upper, mid-, or lower gut [75]. Gut microbiota may be infused either into small intestine (oral capsules, nasoduodenal tube), or into the large intestine (via colonoscopy or enema formulations) [76,77,78]. The oral intake of fecal material through a capsule has been found to be an effective approach in FMT [79]. Capsules containing freeze-dried feces can be administered orally, bypassing the need for invasive procedures and complicated logistics of having the donor and the recipient repeatedly visit the clinic on the same day. Similar to the oral capsule approach, the infusion of the fecal suspension via colonoscopy or retention enema has been also shown to be safe and effective [56]. Moreover, FMT through a mid-gut transendoscopic enteral tube (TET) is a convenient and safe procedure without any negative effects on patients’ quality of life [78]. Among all delivering methods, NDT-delivered (nasoduodenal tube) FMT is often preferred in clinical practice, as it requires the least processing of fecal samples, which can be administered fresh on the day of donation. The procedure is usually preceded by an intestinal lavage. This approach ensures the highest possible transfer of viable aerobic and anaerobic microbial strains and makes sure that both the small and the large intestinal microbiota are reshaped via FMT.

## 4. Clinical Applications of FMT in Human Diseases

Several studies have investigated FMT as a potential therapeutic approach for the treatment of various diseases, based on its acceptable safety profile and promising clinical efficacy. FMT has been clinically tested for a multitude of digestive and extra-digestive disorders associated with gut dysbiosis, such as cardiovascular diseases, metabolic dysfunction-associated steatotic liver disease (MASLD), metabolic syndrome, insulin resistance, neuropsychiatric and autoimmune diseases, chronic acne, renal and biliary lithiasis, and many others.

In the European consensus conference on FMT applications in clinical practice, it was suggested that the only clinical condition with sufficient scientific evidence for beneficial effects of FMT is CDI, a gut dysbiosis-associated disease [80]. Significant clinical experience has been accumulated with the use of FMT for the treatment of recurrent or refractory CDI, and FMT is currently an FDA-approved (Food and Drug Administration) treatment modality for severe and recurrent CDI. FMT has been successful for the treatment of recurrent CDI with an excellent safety profile according to several cohort studies, randomized clinical trials, reviews, and meta-analyses [63,81,82,83,84,85,86]. Beyond CDI, FMT has proven to be beneficial for several other gastrointestinal disorders, including constipation, IBD, and IBS [87]. In more detail, FMT has been a successful therapeutic approach for patients with active ulcerative colitis, as it has been shown to induce remission without any significant adverse events [88,89,90]. In the field of IBD, two systematic reviews for FMT use in Crohn’s disease, analyzing the safety and efficacy of this approach in these difficult-to-treat patients, concluded that FMT may lead to satisfying overall clinical remission rates without any safety risks [91,92]. Of note, baseline gut microbiota abnormalities may serve as an indicator of potential responders to FMT in patients with Crohn’s disease according to several control trials [93,94]. An additional area of successful FMT clinical application has been IBS. FMT has shown the potential to induce significant symptom relief and improvement in fatigue and quality of life in patients with IBS, as a result of gut dysbiosis amelioration [95,96,97]. Nevertheless, more studies are needed to fully elucidate the link between FMT and IBS symptoms [98].

**Figure 1:**
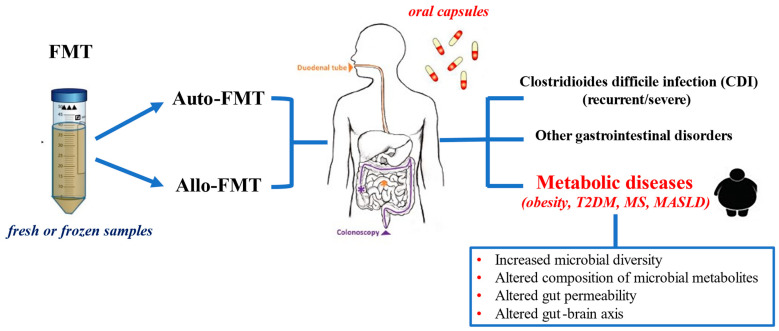
Basic principles and clinical applications of FMT in humans.

Figure 1 illustrates in a schematic way the basic principles and clinical applications of FMT, including metabolic diseases.

## 5. The Role of FMT in Obesity

### 5.1. Experimental Data (Animal Models)

The gut microbiota in obese individuals is more competent in harvesting energy from nutrition, since it makes indigestible fibers digestible and thus stimulates energy uptake [99]. By digesting nutrients, the microbiota stimulates the production of important metabolites such as SCFAs, increases glucagon-like peptide 1 (GLP-1) release and modulates the bile acid pathways, facilitating fat digestion [100]. Furthermore, rat models have shown that some microbiota can increase gut permeability and increase the resorption of endotoxins, thereby accentuating chronic subclinical inflammation, which mediates increased cardiometabolic risk [100]. In this context, FMT may be beneficial for weight loss and metabolic function by means of altering the increased energy harvest of obese patients and modulating gut microbiota in the direction of eliminating harmful species [101]. Considering that obesity has been associated with specific alterations in the gut microbiota with a possible pathogenetic role for weight dysregulation, manipulation of gut microbiota by FMT has been proposed as a potential therapeutic approach for restoring gut dysbiosis and treating obesity [102,103,104,105].

The possible relationship between obesity and gut microbiota was initially suggested, when studies in germ-free mice showed a significant increase in their body fat content despite lower caloric intake after FMT derived from normal mice [106]. A similar effect has been also observed in humans. Gut microbiota alterations have been associated with several complications in overweight and obese humans [107]. Overall, several studies have associated obesity with specific gut bacteria, such as genera of *Bifidobacterium, Lactobacillus,* and *Akkermansia*. The supplementation with *Akkermansia muciniphila* has been shown to improve metabolic outcomes in overweight and obese humans [108], while *Lactobacillus* and *Bifidobacterium* have important beneficial effects on gut microbiota balance and metabolic homeostasis [109]. All these findings clearly suggest that obesity-specific gut microorganisms may be related to several metabolic mechanisms of obesity.

Studies in mice have shown that Auto-FMT may potentiate the weight loss effects of a moderate caloric restriction (CR) in the short term, by decreasing feed efficiency and increasing adipose tissue lipolysis [110]. In this animal study, although FMT produced a significant increase in bacterial diversity, it did not modify gut microbiota composition at phyla and genera levels compared to CR, and only significant increases in *Bifidobacterium* and *Blautia* genera could be observed, suggesting that the anti-obesity effects of Auto-FMT may be related more to changes in bacterial richness rather than in wide phylum levels. These data suggested that Auto-FMT might represent a promising approach for obese patients that do not respond adequately to moderately restrictive diets. An additional experimental study in rats, applying a multiomics approach combining metagenomics and metaproteomics to study microbiota composition and function, has shown that FMT may serve as a complementary weight loss therapy, since it is potent to reverse the adverse effects of antibiotics upon gut microbiota and restore gut microbiota balance, improving host homeostasis and metabolic functions [111]. In the latter study, FMT restored microbiota biodiversity and functionality, suggesting that diet-induced dysbiosis and obesity can be significantly improved by FMT.

### 5.2. Clinical Data (Humans)

Moving to the setting of clinical trials, in a 12-week, double-blind, randomized, placebo-controlled pilot trial investigating the relationship between FMT-achieved gut microbiota modulation and metabolic improvement in obese individuals, the weekly administration of an oral FMT capsule derived from healthy lean donors resulted in gut microbiota engraftment in most obese recipients for at least 12 weeks, but despite successful engraftment, no clinically significant metabolic or weight loss effects were observed [112]. In this study, the primary metabolic outcome was a change in insulin sensitivity between 0 and 6 weeks assessed by euglycemic hyperinsulinemic clamps. Exploratory analyses suggested possible metabolic improvements after FMT among participants with low baseline gut microbiota diversity. These data, and mainly the observation that metabolic and microbiota responses to FMT are highly dependent on a complex host–recipient interaction, suggested that FMT-induced changes in gut microbiota composition alone, without concurrent dietary or other approaches, are rather insufficient to treat or prevent obesity and metabolic disorders in humans. In another double-blinded, randomized, placebo-controlled pilot study with similar design and rationale, 22 metabolically healthy obese patients who received for 12 weeks FMT oral capsules derived from a single lean donor, did not reduce their body weight, although the procedure was safe and induced sustained alterations in the intestinal microbiota and bile acid outcomes that were similar to those of the lean donor [113]. An additional double-blinded, randomized, placebo-controlled, phase 2 trial in severely obese patients with metabolic syndrome, tested the effects of oral FMT application derived from healthy lean donors in the setting of concurrent daily fiber supplementation, and could show an improvement in insulin sensitivity, but only in those obese patients undergoing the FMT approach and supplemented with low-fermentable fibers [114]. In a randomized clinical trial investigating the potential effects of FMT derived from a lean donor on weight loss in obese patients undergoing bariatric surgery, FMT failed to demonstrate any effects on either presurgical or postsurgical weight loss [115]. In the same direction of negative effects upon weight loss, FMT in the form of oral capsules derived from healthy lean donors did not result in significant weight loss in obese adolescents in the Gut Bugs randomized controlled trial, although there was a significant improvement in gut microbial diversity and composition and a small decrease in abdominal adiposity [116].

In 2019, Zhang et al. conducted the first systematic review investigating the impact of Allo-FMT in obesity and metabolic syndrome, and concluded that the procedure was associated with short-term improvements in insulin sensitivity, but had no effect on any other cardiometabolic risk markers including weight loss [117]. This systematic review included three randomized, placebo-controlled studies, in a total of 76 patients with obesity and metabolic syndrome, and found no differences in fasting plasma glucose, hepatic insulin sensitivity, BMI, lipid markers, and fecal SCFA levels after FMT across all studies. Another systematic review and meta-analysis of randomized clinical trials investigating the efficacy of FMT in obesity concluded that FMT may improve selected parameters of metabolic syndrome in obese patients (i.e., glycated hemoglobin HbA1c), but without any appreciable effects on weight loss [118]. Additional reviews summarizing the available evidence on the effects of FMT on obesity and glucose metabolism in humans suggested that FMT derived from lean donors may significantly improve insulin sensitivity in the setting of obesity and metabolic syndrome, but with neutral effects on body weight [119].

While FMT has shown no significant effects on weight loss in the majority of clinical trials, it has been interestingly shown that Auto-FMT in the form of frozen capsules, collected during an initial 6-month weight loss phase and performed in the subsequent weight regain phase, may prevent or attenuate weight regain and preserve glycemic control, in association with specific microbiota signatures. Of note, a green plant-based version of the Mediterranean diet enriched in polyphenols, is able to optimize gut microbiota composition, paving the way for a successful FMT procedure [120].

## 6. The Role of FMT in T2DM and Metabolic Syndrome

### 6.1. FMT with Dietary Intervention in T2DM

Gut microbiota dysbiosis has been related to T2DM pathophysiology and its associated complications, suggesting an interdependent relationship between T2DM and gut microbiota [121]. It is important to note, however, that the evidence supporting the causal nature of this relationship in humans is scarce. In animal studies, FMT derived from human healthy lean donors has been shown to reduce fasting plasma glucose levels, improve lipids and the gut microbiota composition, ameliorate insulin resistance, improve the function of pancreatic islet β cells, and decrease pancreatic tissue inflammation in diabetic mice [122,123].

In humans, there is a paucity of evidence testing the effects of FMT in patients with T2DM without the concomitant administration of antidiabetic medications. A randomized, controlled study in 29 southeast Chinese patients with newly diagnosed T2DM, investigating the metabolic effects of FMT alone, or as an adjunct to metformin in terms of restoring insulin resistance and improving metformin sensitivity, reported that both FMT and the combined approach of FMT plus metformin can improve insulin resistance, adiposity, and gut microbial composition with high colonization and effective engraftment rates ranging between 62.5 and 66.7% after 4 weeks of treatment [124]. Over a longer period of intervention (24 weeks), repeated sessions of FMT derived from lean donors resulted in a significantly increased engraftment of metabolically beneficial microbiota (high abundance of *Bifidobacterium* and *Lactobacillus*) in obese patients with T2DM [125]. These genera have been often investigated for their antidiabetic effects, and their abundance has been inversely correlated with T2DM [126,127]. Another non-blinded, single-arm, FMT intervention study of 12 weeks duration reported a significant decrease in fasting plasma glucose and HbA1c and a significant increase in postprandial c-peptide levels, which was accompanied by a lower abundance of *Rikenellaceae* and *Anaerotruncus* genera, in 17 patients with T2DM compared with 20 healthy controls [128]. The abundance of *Rikenellaceae* has been reported to be associated with diabetes and obesity [129], while the genus *Anaerotruncus* has been associated with several unfavorable metabolic states including glucose intolerance and insulin resistance [130]. In the latter study, there was significant individual variability in response to FMT treatment. FMT responders had significantly higher baseline levels of *Rikenellaceae* and *Anaerotruncus* in their pretreated fecal samples compared to non-responders, which could predict the clinical response to FMT with an area under the curve of 0.83. These findings suggest that the therapeutic effects of FMT can be determined by baseline gut microbiota composition and highlight the necessity to develop individualized precise treatments for T2DM patients, based on their baseline gut microbial qualitative composition.

With regard to possible synergistic effects of FMT with dietary interventions, an open-label, controlled, observational trial investigating the potential health benefits of combining specific diets with FMT in 13 patients with T2DM, has shown that patients who pursued specifically designed diets enriched with probiotics, prebiotics, and whole-grain products in addition to FMT derived from healthy donors had significant benefits in terms of weight loss, glycemic and blood pressure control, and lipid metabolism, possibly due to a faster intestinal microbiota reconstruction induced by FMT after 3 months of intervention [131]. These findings also revealed rapid increases in metabolically beneficial genera such as *Prevotella*, which have shown a positive correlation with optimal metabolic health [132]. The above data indicate that FMT may work in synergy with fiber-rich dietary interventions accelerating and potentiating the weight loss effect.

### 6.2. FMT in Metabolic Syndrome

In patients with metabolic syndrome, it has been shown that Allo-FMT can result in improved insulin sensitivity, in association with altered gut microbiota composition in both the small and large intestine [133]. According to these data, FMT has no effect on serum bile acid or incretin levels, but increases fecal cholate excretion, suggesting possible effects on postprandial metabolism. In this population of metabolic syndrome, clinical efficacy was most pronounced in recipients with a lower gut microbiota diversity at baseline. Indeed, baseline gut microbiota diversity and composition may affect FMT treatment response, at least in terms of insulin resistance [112,133]. In another randomized, controlled trial in men with metabolic syndrome, nasoduodenal tube-facilitated Allo-FMT derived from lean individuals was associated with improved insulin sensitivity and increased microbial diversity compared to individuals who received Auto-FMT after 6 weeks of intervention [134]. Among changes in gut microbial composition, the increase in *Roseburia* intestinalis and *Eubacterium hallii*, both of which are butyrate-producing microorganisms, was the most notable. Of note, not all participants responded to FMT and the study population was of modest size (*n* = 18), limiting the strength of the evidence [135].

### 6.3. FMT in Metabolic Dysfunction Associated Steatotic Liver Disease

Metabolic dysfunction-associated steatotic liver disease (MASLD) is present in the majority of patients with T2DM and metabolic syndrome. With regard to gut microbiota alterations, MASLD patients have shown an increased intestinal permeability promoting endotoxemia, increased numbers of *γ-Proteobacteria*, and decreased numbers of *Bacteroidetes* [42]. It has been suggested that the gut–brain axis and the severity of MASLD can be altered after FMT in the setting of metabolic syndrome [136]. In a study aiming to assess whether FMT derived from vegan donors would improve hepatic inflammation in patients with MASLD, there was a trend for a lower necro-inflammatory histological score and lower hepatic inflammatory gene expression after FMT [136], both of which are important predictors of progression towards steatohepatitis (MASH) that may culminate in liver cirrhosis. Another small-scale FMT trial in patients with MASLD found that FMT from healthy donors reduced gut permeability, an important trait of metabolic dysfunction linked to many adverse health outcomes, although there was no effect on liver steatosis assessed by magnetic resonance imaging (MRI), underscoring the need to use gold-standard methods for MASLD assessment such as biopsy-derived liver histology [137].

Table 3 summarizes the major studies investigating the use of FMT for the management of obesity, T2DM, and metabolic syndrome in humans.

## 7. Limitations, Critical Perspectives, Areas for Future Research

Overall, FMT is a safe procedure with a very low risk of adverse events according to several systematic reviews and meta-analyses, mainly for the clinical indication of CDI [138,139,140,141]. Minor side effects range from diarrhea and constipation to fever and abdominal pain, all of which are mild and short-termed, whereas the frequency of severe adverse events such as CDI and worsening colitis has been reported to be very low [73,141]. Nevertheless, despite being a generally safe procedure based upon rigorous pre-treatment screening protocols, FMT still has the potential for unforeseen complications as is the case with any medical procedure. FMT is not completely without risks, as there have been sporadic reports of host infections and transmission of multi-resistant bacteria [142]. On the other hand, there are several concerns regarding the long-term safety of FMT, including the possibility of weight gain, IBD flares, manifestation of autoimmune and neurological diseases, and allergies [143,144,145]. Although these risks do exist, they seldomly cause concerns, since there are key recommendations about careful donor selection and screening that improve the safety of FMT and ensure its quality and accessibility for all patients [146].

The main pitfalls related to FMT studies that hamper the interpretation and comparability of their findings comprise the mode of delivery (route of transplant administration), the open-label design or often inevitable unblinding of treatment allocations due to mild gastrointestinal complaints associated with FMT, the processing of fecal samples, donor–recipient interaction and optimal matching, gut microbiota diversity, and concomitant diet and medications [45]. All these factors have been reported to potentially modify the engraftment success and thereby long-term FMT treatment efficacy. The large heterogeneity in clinical outcomes of FMT studies may be attributable to variations in transplantation algorithms, study populations selected, heterogeneous composition of fecal transplants and small sample sizes. The beneficial effects of FMT are likely achieved through pleiotropic mechanisms that may even differ in different clinical conditions. Of note, these effects may be also mediated by other components of the donor fecal transplant (non-bacteria), i.e., the donor’s bacteriophages.

It has been consistently shown that the efficacy of FMT depends on the microbial diversity and composition of donor fecal material, as well as on baseline diversity and gut microbiota composition of the recipient, especially with regard to metabolic outcomes. An important tool to better interpret FMT studies would be a detailed tracking of baseline and post-FMT gut microbial composition with deep metagenomic sequencing of fecal samples, allowing the identification of microbial strains based on specific single-nucleotide variations. Despite the significant advances in the past two decades in quantitative metagenomics, our understanding of the metabolic functions of different bacteria remains poor. How the transplanted “whole-gut” bacteria may adapt to the host environment to affect phenotypic changes is not completely understood and is also challenging to quantify. Studies using a more targeted approach, where one or selected few bacteria strains are inoculated in a new host, can provide better insights into their role leading to a successful microbiota restoration. It is possible, indeed, that a single-strain transfer may be more impactful than entire FMT. Therefore, FMTs enriched by certain strains may hold promise for improved outcomes in the future.

The gut ecosystem resilience to invasion by new species may pose a challenge to successful FMT engraftment and minimize potential metabolic benefits. One potential solution to improve engraftment, sustain reversal of dysbiosis, and further improve metabolic outcomes, would be to apply microbiota-targeted dietary strategies after FMT (i.e., diet enriched with prebiotics). Concomitant diet may indeed alter the response to FMT by independently shaping microbiota composition. Given that the human gut microbiota is a complex ecosystem, the ecological challenges of engraftment such as microbiota resilience, competitive exclusion, and host environmental interactions should be considered when developing future FMT studies. Dietary interventions would be one option to maintain FMT engraftment and enhance metabolic effect. More research is needed to better standardize the optimal fecal microbial preparation, dose–response relationship (grams of donor stool and/or microbial load in the stool), and method of delivery [117].

## 8. Summary and Concluding Remarks

Despite its limitations, FMT is currently one of the most important tools to investigate the causal relationship of gut microbiota with a variety of chronic clinical conditions. Obesity and T2DM are prevalent metabolic disorders, and extensive research has corroborated their association with gut microbiota dysbiosis. There is promising clinical evidence that FMT may effectively reshape host microbiota and thereby promote beneficial effects on hyperglycemia, insulin resistance, and metabolic dysfunction, through mechanisms independent of weight loss.

Although FMT procedures have yielded important mechanistic insights, their use in clinical practice may be limited due to practical constraints in the setting of metabolic diseases. It is important to note that further targeted research is required to uncover the potential efficacy of FMT in metabolic diseases. The intestinal flora is not static but is continuously influenced by various epigenetic and environmental factors. This dynamic nature of the gut microbiota poses challenges for achieving long-term efficacy through FMT. Furthermore, host immunity and lifestyle interfere with long-term engraftment of the donor microbiota. This underscores that donor–recipient matching and selection is crucial, as it might confound the response to FMT in patients with obesity and T2DM. Considering that every individual’s baseline gut microbiota is different and knowledge as to what defines a healthy qualified FMT donor remains limited and incomplete, further research is needed. With sophisticated metabolomics, sequencing, and bioinformatics, the potential mechanisms of action of FMT may be better characterized.

In conclusion, additional research is warranted to better understand the effects of FMT on diabetes- and obesity-related mechanisms. This will enable a comprehensive evaluation of its potential as a therapeutic approach for these epidemic disorders. By considering the influence of various epigenetic factors on the gut microbiota, researchers can enhance our understanding of FMT long-term effectiveness and develop more successful transplantation strategies. The question in the coming decade will be whether targeted microbiota-based treatments, such as FMT with or without added specific bacterial strains, can help to potentiate existing dietary and pharmaceutical therapeutic strategies to improve human metabolic health.

## Figures and Tables

**Table 1:** Exclusion criteria of the European Commission for candidate FMT donors at the preliminary interview.

Exposure to HIV, HBV or HCV, syphilis, human T-lymphotropic virus I and II, malaria, trypanosomiasis, tuberculosis
2. Systemic infection not controlled at the time of donation
3. Use of illegal drugs
4. Risky sexual behavior (anonymous sexual contacts; sexual contacts with prostitutes, drug addicts, individuals with HIV, viral hepatitis, syphilis; work as prostitute; history of sexually transmittable disease)
5. Previous reception of tissue/organ transplant
6. Previous (<12 months) reception of blood products
7. Recent (<6 months) needle stick accident
8. Recent (<6 months) body tattoo, piercing, earring, acupuncture
9. Recent medical treatment in poorly hygienic conditions
10. Risk of transmission of diseases caused by prions
11. Recent parasitosis or infection from rotavirus, Giardia lamblia, and other microbes with gastrointestinal involvement
12. Recent (<6 months) travel in endemic areas of gastrointestinal pathogens
13. Recent (<6 months) history of vaccination with a live attenuated virus, if there is a possible risk of transmission
14. Healthcare workers (to exclude the risk of transmission of multidrug-resistant organisms)
15. Individual working with animals (to exclude the risk of transmission of zoonotic infections)
16. Metabolic and neurological disorders
17. History of IBS, IBD, chronic constipation, celiac disease, other chronic GI disorders
18. History of chronic, systemic autoimmune disorders with GI involvement
19. History of, or high risk for, GI cancer or polyposis
20. Recent appearance of diarrhea, hematochezia
21. History of neurological/neurodegenerative disorders
22. History of psychiatric conditions
23. Overweight and obesity (BMI > 25)
24. Drugs that can impair gut microbiota composition
25. Recent (<3 months) exposure to antibiotics, immunosuppressants, chemotherapy
26. Chronic treatment with proton pump inhibitors

Abbreviations: BMI: body mass index; GI: gastrointestinal; HBV: hepatitis B virus; HCV: hepatitis C virus; HIV: human immunodeficiency virus; IBD: inflammatory bowel disease; IBS: irritable bowel syndrome.

**Table 2:** Exclusion criteria for candidate FMT donors at the donation day interview.

New-onset gastrointestinal signs and symptoms, for example, diarrhea, nausea, vomiting, abdominal pain, and jaundice
2. New-onset illness or general signs as fever, throat pain, swollen lymph nodes
3. Use of antibiotics or other drugs that may impair the gut microbiota, new sexual partners or travels abroad since the last screening
4. Recent ingestion of a substance that may have harmful effects for the recipients
5. Travel in tropical areas, contact with human blood (sting, wound, piercings, tattoos), and sexual high-risk behavior
6. Diarrhea (more than three loose or liquid stools per day) among members of the family (including children) within 4 weeks prior to donation

**Table 3:** Major clinical studies assessing FMT application for the improvement of obesity and metabolic parameters in the setting of obesity, T2DM, and metabolic syndrome.

Study Design	Study Population	FMT Intervention and Duration	Major Findings	Reference
Randomized, double-blinded, placebo-controlled, pilot study	*n* = 22 metabolically healthy obese patients	Oral capsules derived from a single lean donor (induction dose of 30 capsules at week 4 and maintenance dose of 12 capsules at week 8), follow-up of 26 weeks	No effects on BMI and GLP1 postprandial response, sustained changes in gut microbiota and bile acid profiles resembling the lean donor profile, safe procedure, well-tolerated, no adverse events	[113]
Randomized, double-blinded, placebo-controlled trial (Gut Bugs Trial)	*n* = 87 obese adolescents aged 14–18 years old, New Zealand	Single course of oral FMT capsules derived from 4 healthy lean donors, follow-up of 26 weeks	No effects on weight loss, insulin sensitivity, liver function, lipid profile, inflammatory markers, blood pressure, total body fat %, gut health and health-related quality of life, reduction of abdominal adiposity, resolution of metabolic syndrome in post hoc analyses, maintained shift in gut microbiota profiling for up to 12 weeks, no serious adverse events	[116]
Randomized, double-blinded, placebo-controlled pilot trial	*n* = 24 obese patients with mild to moderate insulin resistance, treated at a single academic medical center of US	Weekly oral FMT capsules derived from healthy lean donors Treatment for 6 weeks Follow-up of 12 weeks	No effects on insulin sensitivity, body composition and other secondary metabolic outcomes, variable engraftment of donor gut microbiota maintained for at least 12 weeks, no serious adverse events	[112]
Randomized, double-blinded, placebo-controlled, parallel four-arm, phase 2 clinical trial	*n* = 70 patients with severe obesity (BMI > 40) and metabolic syndrome 4 groups compared: FMT + HF (*n* = 17) FMT + LF (*n* = 17) HF (*n* = 17) LF (*n* = 19)	Single-dose oral FMT capsules derived from healthy lean donors combined with daily fiber supplementation of high or low fermentability Treatment for 6 weeks	Improved insulin sensitivity only in FMT + LF group, effects independent of diet and medications, but associated with altered microbial ecology, improved enteroendocrine responses after OGTT and increased engraftment of donor microbes, safe and well-tolerated intervention	[114]
Randomized, placebo-controlled clinical trial DIRECT PLUS weight loss trial (Dietary Intervention Randomized Controlled Trial Polyphenols-Unprocessed)	*n* = 90 abdominally obese or dyslipidemic Israel patients undergoing weight loss with a 6-month Mediterranean diet, follow-up over the weight regain phase (6–14 months)	Diet-modulated Auto-FMT in the form of 100 frozen capsules performed over the weight regain phase, after the 6-month weight loss phase	Attenuated weight regain, waist circumference gain, and insulin rebound only in the group having lost weight with a polyphenol-enriched, green plant-based version of Mediterranean diet No adverse events reported	[120]
Randomized, double-blinded, placebo-controlled, multi-center trial	*n* = 41 severely obese adults undergoing bariatric surgery at two centers in Finland	Allo-FMT derived from a lean donor vs. Auto-placebo performed by gastroscopy into duodenum, bariatric surgery (LRYGB or LSG) performed 6 months after baseline FMT intervention, follow-up of 18 months	No effects on total weight loss % at 6 months (prior to surgery) or at 18 months (post bariatric surgery) No effect of FMT in potentiating the bariatric surgery weight loss effect	[115]
Non-blinded, single-arm, FMT intervention trial (prospective cohort study)	*n* = 17 patients with T2DM	FMT derived from healthy lean donors, follow-up of 12 weeks	Reduced HbA1c, fasting plasma glucose and uric acid levels after FMT, increased postprandial c-peptide levels, pretreated fecal abundance of *Rikenellaceae* and *Anaerotruncus* genera may predict an enhanced metabolic response to FMT	[128]
Randomized, double-blinded, parallel 3-arm, placebo-controlled trial	*n* = 61 obese patients with T2DM 3 groups: FMT + lifestyle intervention FMT alone Sham transplantation	FMT derived from healthy lean donors ± lifestyle intervention Repeated sessions of FMT Follow-up of 24 weeks	Sustained engraftment of lean donor microbiota after repeated FMTs, increased fecal abundance of *Lactobacillus* and *Bifidobacterium* species, improved lipid profile and reduced liver stiffness 24 weeks after combined FMT and lifestyle intervention	[125]
Randomized, open-label, controlled clinical trial	*n* = 16 patients with T2DM (*n* = 13 completed the study)	Oral FMT (capsules) derived from young healthy donors administered on a weekly basis for 3 weeks, concomitant dietary intervention with dietary formulations rich in prebiotics, probiotics, and whole-grain products, follow-up of 3 months	Accelerated changes in gut microbiota with diet + FMT vs. diet alone, improved blood glucose and blood pressure control, increased fecal abundance of *Bifidobacterium* and decreased abundance of sulfate-reducing bacteria	[131]
Randomized, controlled, prospective study	*n* = 31 Chinese patients with newly diagnosed T2DM	FMT derived from healthy donors delivered via nasojejunal feeding tubes ± metformin, follow-up of 4 weeks	Improved insulin resistance, BMI, fasting, and postprandial glucose levels and HbA1c, enhanced engraftment of donor-associated microbiota	[124]
Randomized, double-blinded, placebo-controlled trial	*n* = 18 male obese Caucasian subjects with metabolic syndrome	Allo-FMT derived from healthy lean, age-matched, male donors (small intestinal infusion via gastroduodenoscopy) vs. Auto-FMT (serving as placebo), follow-up of 6 weeks	Increased peripheral insulin sensitivity assessed by clamps and elevated levels of butyrate-producing fecal bacteria at 6 weeks	[134]
Randomized, double-blinded, controlled trial	*n* = 38 male obese Caucasian subjects with metabolic syndrome	Allo-FMT derived from healthy lean donors (infusion via nasoduodenal tube) vs. Auto-FMT (serving as placebo), Allo-FMT repeated at 6 weeks, follow-up of 18 weeks	Transient short-term beneficial effect of Allo-FMT on peripheral insulin sensitivity assessed by clamps at 6 weeks, metabolic response associated with plasma metabolite and fecal microbial changes and predicted based on baseline gut microbiota diversity and composition	[133]
Randomized, double-blinded, controlled trial	*n* = 21 patients with NAFLD	Allo-FMT derived from a healthy lean donor vs. Auto-FMT delivered endoscopically into distal duodenum, follow-up of up to 6 months post-FMT	No effects on insulin resistance and MRI-assessed hepatic fat content, reduced small intestinal permeability in those with elevated gut permeability at baseline at 6 weeks after Allo-FMT	[137]
Randomized, double-blinded, controlled, proof-of-concept trial	*n* = 21 obese subjects with ultrasound-confirmed hepatic steatosis	Allo-FMT derived from lean vegan donor vs. Auto-FMT (serving as placebo) performed 3 times at 8-week intervals, liver biopsy performed at baseline and at 24 weeks	Trend towards reduced necro-inflammatory burden in liver histology, reduced hepatic gene expression involved in inflammation and lipid metabolism, beneficial changes in gut microbiota composition and plasma metabolites	[136]

Abbreviations: Allo-FMT: allogenic fecal microbiota transplantation; Auto-FMT: autologous fecal microbiota transplantation; BMI: body mass index; FMT: fecal microbiota transplantation; GLP1: glucagon-like peptide 1; HbA1c: glycated hemoglobin A1c; HF: high-fermentable fiber; LF: low-fermentable fiber; LRYGB: laparoscopic Roux-en-Y gastric bypass; LSG: laparoscopic sleeve gastrectomy; MRI: magnetic resonance imaging; NAFLD: non-alcoholic fatty liver disease; OGTT: oral glucose tolerance test; T2DM: type 2 diabetes mellitus; US: United States.
